# Internationale Erkenntnisse zur Sexualaufklärung: 15 Jahre im Rückblick

**DOI:** 10.1007/s00103-026-04219-5

**Published:** 2026-03-24

**Authors:** Katherine Watson, Volker Schmidt-Cox, Johanna Marquardt

**Affiliations:** 1 Universität Leiden, Leiden, Niederlande; 2 Referat Sexualaufklärung, Bundesinstitut für öffentliche Gesundheit (BIÖG), Köln, Deutschland; 3 WHO Kollaborationszentrum für sexuelle und reproduktive Gesundheit, Bundesinstitut für öffentliche Gesundheit (BIÖG), Maarweg 149–161, 50825 Köln, Deutschland

**Keywords:** Sexualerziehung, Sexuelle Bildung, Kinder, Jugendliche, Junge Menschen, Sexuality education, Sexual development, Children, Adolescents, Young people

## Abstract

**Einleitung:**

Umfassende Sexualaufklärung (Comprehensive Sexuality Education, CSE) ist zentral für die gesunde Entwicklung junger Menschen, die Förderung gesunder Beziehungen und Geschlechtergerechtigkeit. Seit der Veröffentlichung der WHO-Standards 2010 wurde die Evidenzbasis deutlich erweitert und neue Diskurse haben an Bedeutung gewonnen. Ziel dieser Arbeit sind ein systematischer Überblick über die seit 2010 publizierte Evidenz zu schulischer CSE in Europa sowie die Identifikation neuer thematischer Entwicklungen.

**Methoden:**

Im Rahmen des Scoping-Reviews erfolgten strukturierte Literaturrecherchen in PubMed, Google Scholar sowie in der Fachzeitschrift *Sex Education*. Die Suchbegriffe kombinierten Varianten von „comprehensive sexuality education“, „sex education“, „school-based“, „adolescence“, „sexual development“ und „Europe“ sowie Begriffe zur kindlichen und jugendlichen sexuellen Entwicklung. Der Suchzeitraum umfasste den 01.01.2010 bis 25.02.2025. Zur Stärkung der europäischen Repräsentation wurden ergänzend Expertinnen und Experten konsultiert. Es folgten Screening, Evidenzerfassung und Validierung der Synthese.

**Ergebnisse:**

Die Evidenz belegt positive Effekte von CSE auf sozialemotionales Lernen, gesunde Beziehungen, Geschlechtergerechtigkeit und Gewaltprävention. Zunehmend betont werden rechtsbasierte, inklusive und diversitätssensible Ansätze sowie die Berücksichtigung digitaler Lebenswelten. Gleichzeitig zeigen sich wachsende gesellschaftliche Widerstände.

**Diskussion:**

Die Analyse verdeutlicht eine inhaltliche Weiterentwicklung von CSE, die bei der Überarbeitung der Standards evidenzbasiert berücksichtigt werden sollte, um Aktualität und Relevanz im europäischen Kontext sicherzustellen.

**Zusatzmaterial online:**

Zusätzliche Informationen sind in der Online-Version dieses Artikels (10.1007/s00103-026-04219-5) enthalten.

## Abstract (en)

**Introduction:**

Comprehensive sexuality education (CSE) is central to the healthy development of young people and, promoting healthy relationships and gender equality. Since the publication of the WHO Standards in 2010, the evidence base has expanded considerably and new discourses have gained importance. The aims of this study are to provide a systematic overview of the evidence on school-based CSE published since 2010 and to identify emerging thematic developments.

**Methods:**

As part of a scoping review, structured literature searches were carried out in PubMed, Google Scholar, and the journal *Sex Education*. Search terms combined variations of “comprehensive sexuality education,” “sex education,” “school-based,” “adolescence,” “sexual development,” and “Europe” as well as terms related to child and adolescent sexual development. The search period covered 1 January 2010 to 25 February 2025. To strengthen European representation, additional references were solicited from members of the expert group. This was followed by screening, evidence extraction, and validation of the synthesis.

**Results:**

The evidence demonstrates positive effects of CSE on social-emotional learning, healthy relationships, gender equality, and violence prevention. Increasing emphasis is placed on rights-based, inclusive, and diversity-sensitive approaches as well as on addressing digital realities. At the same time, growing societal resistance is evident.

**Discussion:**

The analysis highlights a substantive evolution of CSE that should be considered in the evidence-based revision of the Standards to ensure their relevance and applicability within the European context.

## Einleitung

Eine umfassende Sexualaufklärung (Comprehensive Sexuality Education, CSE) ist ein Eckpfeiler für die Entwicklung von Kindern und Jugendlichen und legt den Grundstein für Gesundheit und Wohlbefinden sowie für lebenslange gesunde Beziehungen und Gleichstellung der Geschlechter. CSE wird weltweit als wichtige Strategie zur Förderung positiver Ergebnisse in der sexuellen Entwicklung zugesprochen und im International Technical Guidance on Sexuality Education (ITGSE) wie folgt definiert:

„… [es handelt sich um] einen lehrplanbasierten Lehr- und Lernprozess hinsichtlich der kognitiven, emotionalen, körperlichen und sozialen Aspekte von Sexualität. Dieser zielt darauf ab, Kindern und Jugendlichen Kenntnisse, Fähigkeiten, Einstellungen und Werte zu vermitteln, die sie befähigen, ihre Gesundheit, ihr Wohlbefinden und ihre Würde zu verwirklichen sowie respektvolle soziale und sexuelle Beziehungen aufzubauen, sowie zu reflektieren, wie sich ihre Entscheidungen auf ihr eigenes Wohlbefinden und das anderer auswirken, und ihre Rechte ihr Leben lang zu verstehen und zu schützen“ [1].

Seit den 1960er-Jahren sind in Europa bemerkenswerte Fortschritte bei der Implementierung von CSE-Programmen in Schulen zu verzeichnen, auch wenn die Fortschritte heterogen sind. Umfang, Qualität und Umsetzung der Schulprogramme variieren stark, sowohl national als auch zwischen den 53 Ländern, die dem Regionalbüro für Europa der Weltgesundheitsorganisation (WHO/Europa; [2]) angehören. Als Reaktion darauf hat das WHO-Kollaborationszentrum für sexuelle und reproduktive Gesundheit, angesiedelt im Bundesinstitut für Öffentliche Gesundheit (WHO CC des BIÖG), in Zusammenarbeit mit der Europäischen Expertengruppe für Sexualaufklärung (hier kurz: „Expertengruppe“) und der WHO/EUROPA im Jahr 2010 die „Standards für Sexualaufklärung in Europa: Ein Rahmen für politische Entscheidungsträger, Bildungseinrichtungen, Gesundheitsbehörden, Experten und Expertinnen“ (kurz: die „Standards“) entwickelt und veröffentlicht. Die Standards wurden inzwischen in 15 Sprachen übersetzt und werden im europäischen Raum angewendet.

In den Jahren seit der Veröffentlichung der Standards hat sich die internationale wissenschaftliche Grundlage für CSE signifikant vergrößert. Heute zeigen die vorhandenen Erkenntnisse deutlicher denn je, dass CSE das Potenzial hat, die Gesundheit und das Wohlbefinden von Kindern und Jugendlichen in vielerlei Hinsicht positiv zu beeinflussen und zur sozialen und emotionalen Entwicklung beizutragen. Gleichzeitig hat mit dem Wachstum der Evidenzbasis für CSE auch der Widerstand sowohl von staatlichen als auch von nichtstaatlichen Akteurinnen und Akteuren zugenommen [3]. Fachkräfte aus Pädagogik, Wissenschaft, Interessensvertretung und Sozialarbeit in ganz Europa sehen sich starkem Widerstand ausgesetzt, der vor allem von Online-Kampagnen zur Untergrabung nationaler CSE-Programme, oft mit gezielten Falschdarstellungen, bis hin zu Morddrohungen gegen einzelne Personen reicht.

Angesichts der aktuellen globalen Herausforderungen erkannten das WHO CC des BIÖG und die Expertengruppe die Notwendigkeit, die Standards zu überarbeiten, um sicherzustellen, dass sie eine glaubwürdige, umfassende und maßgebliche Referenz für CSE bleiben. Zu diesem Zweck beauftragte das WHO CC des BIÖG ein Scoping-Review, das den Zeitraum seit der Veröffentlichung der Standards (2010) bis Januar 2025 abdeckt und zusätzlich von einer Expertengruppe präzisiert und validiert wird. Die Forschungsfragen lauten: Welche neue Evidenz zur schulischen Sexualaufklärung wurde seit 2010 publiziert und welche thematischen Schwerpunkte beziehungsweise emergenten Themen lassen sich in der aktuellen Literatur erkennen?

## Methoden

### Schritt 1: Festlegung des Umfangs.

Die Ziele, Abgrenzungen und prioritären Themen des Reviews wurden zu Beginn in Konsultation mit der Expertengruppe präzisiert. In diesem Schritt wurden die Forschungsfragen wie folgt formuliert: 1) Welche neuen wissenschaftlichen Erkenntnisse in der CSE-Forschung wurden seit der Veröffentlichung der Standards 2010 gewonnen? 2) Welche neuen Diskurse haben an Bedeutung gewonnen?

### Schritt 2: Literatursuche.

Gezielte Suchen wurden in der Datenbank PubMed und über Google Scholar durchgeführt. Eine weitere wichtige Quelle auf dem Gebiet der CSE war die renommierte Fachzeitschrift *Sex Education*. Die verwendeten Suchbegriffe kombinierten Varianten von: „comprehensive sexuality education“, „sex education“, „school-based“, „adolescence“, „sexual development“ und „Europe“. Darüber hinaus wurden Google-Scholar-Suchen zur Verbindung von kindlicher und jugendlicher sexueller Entwicklung mit CSE durchgeführt, unter Verwendung der Begriffe: „child“, „adolescent“, „sexual development“, „sex education“ und „sexuality education“. Der Untersuchungszeitraum für alle Suchen wurde auf den 01.01.2010 bis 25.02.2025 festgelegt.

### Schritt 3: Ergänzung der Literatur.

Die Literatursuche lieferte weniger Studien aus Europa als in der weltweit publizierten Literatur. Um die regionale Repräsentation zu erhöhen, wurde eine Umfrage unter den Mitgliedern der Expertengruppe durchgeführt, in der zusätzliche Referenzen aus europäischen Kontexten angefragt wurden. Daraus resultierten 32 weitere Referenzen in Englisch, Französisch, Deutsch, Spanisch und Portugiesisch. Gleichzeitig wurden Referenzlisten der in Schritt 2 identifizierten systematischen Reviews gescreent. Durch die Schritte 2 und 3 konnten insgesamt 242 Studien identifiziert werden.

### Schritt 4: Screening und Erfassung der Evidenz.

Studien aus allen Referenzen wurden gescreent und erfasst, wenn sie die folgenden Einschlusskriterien erfüllten:

- behandelten schulische CSE-Programme,
- untersuchten CSE-Ergebnisse, Implementierung oder Innovation oder stellten einen Bezug zwischen kindlicher/jugendlicher sexueller Entwicklung und CSE her und
- waren peer-reviewte Studien, systematische Reviews oder hochwertige graue Literatur.

Ausgeschlossen wurden Studien, die außerschulische oder hybride CSE-Programme behandelten oder keinen Bezug zu CSE bzw. zur sexuellen Entwicklung von Kindern und Jugendlichen aufwiesen. 90 Referenzen erfüllten die Einschlusskriterien und wurden in einer Übersichtsmatrix erfasst, die Studientyp, Kontext und Relevanz dokumentierte. Etwa 53 dieser Referenzen werden in diesem Review direkt zitiert; die übrigen dienten zur Entwicklung des thematischen Rahmens.

### Schritt 5: Synthese und Validierung durch Expertinnen und Experten.

Die Ergebnisse wurden in Excel mittels narrativer, thematischer Analyse synthetisiert. Die Themen wurden induktiv abgeleitet, durch Diskussionen zwischen den Autorenschaften dieses Artikels weiterentwickelt und durch die Expertengruppe validiert. Ein persönliches Treffen der Expertengruppe im August 2024 diente als formelle Konsultation und trug zur weiteren Präzisierung und Validierung der entstehenden Themen bei.

## Ergebnisse

### CSE beeinflusst zahlreiche Gesundheits- und weitere Bereiche

Früher wurde die Wirksamkeit von CSE in Studien primär durch Indikatoren für die öffentliche Gesundheit gemessen – insbesondere für die sexuelle und reproduktive Gesundheit. Dazu gehörten die Senkung der Teenagerschwangerschaftsrate sowie von HIV und anderen sexuell übertragbaren Infektionen (STIs; [4, 5]). Dementsprechend eigneten sich die Inhalte von CSE gut dazu, Risiken zu verringern. CSE wird in vielen Kontexten in Westeuropa und weltweit als Reaktion des Staates auf neue Phänomene im Bereich der öffentlichen Gesundheit und der Gesellschaft verstanden, darunter das Auftreten und die Verbreitung von HIV, die Verfügbarkeit der Antibabypille und die Entkriminalisierung von Schwangerschaftsabbrüchen [2, 6, 7].

In den letzten 10 bis 15 Jahren wurden jedoch die Forderungen lauter, die Forschung zu erweitern [4, 5, 8–10]. Im Jahr 2016 wies die Expertengruppe darauf hin, dass „die aktuelle Literatur zeigt, dass sich die Bewertungskriterien überwiegend auf die Auswirkungen auf die öffentliche Gesundheit konzentrieren“ – jedoch häufig zulasten positiverer Indikatoren für Sexualität sowie für die Entwicklung von Kindern und Jugendlichen [11].

Als Reaktion auf die lückenhafte Evidenz und die Forderungen nach Anerkennung des breiteren Potenzials von CSE hat die Wissenschaft in den letzten 10 Jahren damit begonnen, die Forschung zu erweitern [4, 8, 12]. Bei der Suche nach Studien, die andere CSE-Ergebnisse als ausschließlich die Verringerung von STIs oder Schwangerschaften gemessen haben, identifizierten Goldfarb und Lieberman [8] in ihrer systematischen Übersicht 48 Artikel, davon 39 aus den USA. Sie gruppierten diese Studien je nach den verwendeten Messungen in mehrere Kategorien:

- Wertschätzung von Vielfalt,
- Prävention von Gewalt in Beziehungen und beim Dating,
- gesunde Beziehungen,
- Prävention von Kindesmissbrauch und
- sozialemotionales Lernen und digitale Kompetenz [8].

Neben der Erweiterung der Messmethoden haben manche Studien auch versucht, sich von einem „Risikoansatz“ zu lösen und sich stattdessen auf die positiven Auswirkungen der CSE auf das Leben junger Menschen zu konzentrieren. So gab beispielsweise die UNESCO 2022 eine Studie in Auftrag, deren Ergebnisse in Kürze veröffentlicht werden und das Potenzial schulischer CSE zur Verbesserung der Beziehungen von Lernenden zu ihren Eltern erkunden sollen; es wurden Jugendliche und ihre Beziehungspartnerinnen und -partner in 6 Ländern untersucht.

Expertinnen und Experten forderten außerdem ganzheitlichere Messmethoden, die sich von randomisierten Kontrollstudien (RCTs) als Goldstandard entfernen und stattdessen die Bedeutung qualitativer Belege sowie der Perspektiven junger Menschen stärker in den Blick nehmen:

„Mixed methods-Forschung – unter Einbeziehung von RCTs und damit verbundenen qualitativen Untersuchungen – ist unerlässlich, um die vielen Facetten der Wirksamkeit zu verstehen, wie sie in Bildungsumgebungen und im Leben junger Menschen zum Tragen kommen. Ebenso lässt sich der Begriff ‚Wirksamkeit‘ je nach den Zielen eines bestimmten Programms oder einer bestimmten Maßnahme unterschiedlich auslegen. Beispielsweise gibt es im Bildungsbereich übergeordnete Ziele, die weit über die Bewertung eines einzelnen Programms über einen kurzen Zeitraum (z. B. ein Schuljahr) hinausgehen“ [4].

### Die kritischen Entwicklungsphasen bieten Chancen für CSE

Seit 2010 hat die Forschung ein zunehmendes Interesse daran gezeigt, wie sexuelle und reproduktive Gesundheit und Rechte (SRHR) und insbesondere CSE-Programme für jüngere Altersgruppen differenziert untersucht werden sollten. Verschiedene Studien haben sich mit den sich überschneidenden Kategorien „sehr junge Jugendliche“ im Alter von 10 bis 14 Jahren [13–16] und „Grundschulkinder“ im Alter von 5 bis 12 Jahren befasst [17–19]. Wenige Studien widmeten sich Kindern im Vorschul- oder Kindergartenalter, obwohl Untersuchungen in Finnland mit Kindern im Vorschulalter (1–6 Jahre) zeigen, dass Sexualität schon in den frühen Jahren Teil des lebenslangen Entwicklungsprozesses ist (siehe Infobox; [20, 21]).

Um die Bedeutung der entwicklungsgerechten CSE für Kinder und Jugendliche aller Altersgruppen zu betonen und das Potenzial jedes einzelnen Entwicklungszeitraums für bestimmte Ergebnisse zu veranschaulichen, stützt sich die Forschung auf Studien zur Entwicklung von Kindern und Jugendlichen. In ihrer Vorschul- und Grundschulzeit durchlaufen Kinder eine Reihe kognitiver, neurologischer, sozialer, emotionaler und körperlicher Entwicklungen, von einer gesteigerten Fähigkeit zum abstrakten Denken bis hin zu einem besseren Verständnis von Stereotypen, Geschlechterrollen und Selbstidentifikation [17, 20]. All das trägt zur sexuellen Entwicklung bei. Diese rasch aufeinanderfolgenden Entwicklungsphasen bieten Möglichkeiten, die Gesundheit und das Wohlbefinden sowohl in der Kindheit und Jugend als auch über den gesamten Lebensweg zu verbessern, unter anderem in Bezug auf [8, 17, 22–25]:

- die Förderung von Fähigkeiten, die das lebenslange Lernen stützen;
- die Vermittlung von Wissen und zur Prävention von Missbrauch und zum Umgang mit (sexualisierter) Gewalt;
- die Verringerung von Mobbing in Schulen und im Internet;
- die Senkung des Stressniveaus und die Steigerung der Selbstwirksamkeit;
- die Verbesserung der Bewältigungsfähigkeiten sowie anderer sozialer und emotionaler Fähigkeiten;
- die Verbesserung der sexuellen und reproduktiven Gesundheit im späteren Leben;
- die Förderung der Gleichstellung der Geschlechter;
- den Beitrag zu aktivem sozialen Engagement.

Besonderes Augenmerk erhielt das Potenzial von CSE-Programmen, zur Bildung gerechter Geschlechternormen beizutragen, die sich bereits ab dem Alter von 5 Jahren entwickeln [17, 26]. Es gibt Hinweise darauf, dass die frühen Schuljahre der beste Zeitpunkt sind, sexualitätsbezogene Themen einzuführen, und dass die Auseinandersetzung in der CSE mit Geschlechter- und Machtdynamiken bei einer Reihe anderer Gesundheits- und Wohlbefindensindikatoren zu besseren Ergebnissen führt [8, 9].

„Diese Untersuchung legt nahe, dass jüngere Kinder nicht nur in der Lage sind, über sexualitätsbezogene Themen zu diskutieren, sondern dass die frühen Schuljahre tatsächlich der beste Zeitpunkt sein könnten, um Themen im Zusammenhang mit sexueller Orientierung, Geschlechtsidentität, Geschlechtsausdruck, Geschlechtergleichstellung und sozialer Gerechtigkeit in Bezug auf die LGBTQ-Gemeinschaft einzuführen, noch ehe hetero- und cisnormative Werte und Annahmen tiefer verwurzelt und weniger veränderbar werden. Kinder lernen geschlechtsspezifisches Rollenverhalten in jungen Jahren vor allem durch die Beobachtung ihrer Familienmitglieder“ [8].

Die Forschung weist darauf hin, dass sich mit CSE in der frühen Adoleszenz weitere positive Ergebnisse erzielen lassen, darunter eine geringere Akzeptanz von Vergewaltigungsmythen^1^ im späteren Leben [27], einen Rückgang sexueller Gewalt [8, 19], die Entwicklung und Aufrechterhaltung gesunder Beziehungen während des gesamten Lebens (UNESCO, in Vorbereitung), prosoziales Verhalten wie Freundlichkeit, bereitwilliges Teilen und Empathie und damit verbundene höhere Bildungsabschlüsse [28, 29] sowie sexuelles Wohlbefinden [30].

### CSE sollte auf die Entwicklung von Kindern und Jugendlichen abgestimmt sein

Die Literatur zur Entwicklung von Kindern und Jugendlichen erkennt die Notwendigkeit an, sich von einem ausschließlich auf den Koitus konzentrierten Verständnis von Sexualität zu lösen und ein Verständnis dafür zu entwickeln, Sexualität in jeder Lebensphase als etwas Eigenständiges zu betrachten. Cacciatore und Kollegen [31] haben „Stufen der Sexualität“ formuliert, die einen Rahmen für die sexuelle Entwicklung von der Geburt bis zur Adoleszenz bieten. Die Stufen reichen von „Ich bin wunderbar“ (Entdeckung des eigenen Körpers und Interesse an den Unterschieden zwischen den Geschlechtern: Säuglings- und Kleinkindalter) bis hin zu „Reife für die Liebe“ (Bereitschaft für die erste sexuelle Erfahrung: mittlere bis späte Adoleszenz). Die Autorenschaft vertritt zwar die Auffassung, dass die Entwicklung über die einzelnen Stufen nicht unbedingt linear verläuft, doch müssen die „Ziele“ jeder Stufe erreicht werden, um zur nächsten übergehen zu können. Wichtig ist, dass Kinder und Jugendliche im Vorhinein Informationen über jede Stufe erhalten.

Obwohl die empirischen Forschungen zu sexueller Entwicklung und CSE überschaubar sind, zeigen einige Studien aus dem europäischen Kontext, dass grundlegende, im Säuglingsalter entwickelte sexualitätsbezogene Kompetenzen in der Kindheit und Jugend weiter ausgebaut werden. Eine Studie mit finnischen Erzieherinnen und Erziehern ergab, dass die emotionalen und körperlichen Grundlagen einer gesunden Sexualität bereits im Alter von einem Jahr entstehen [21]. Für die sexuelle Entwicklung von Jugendlichen ist eine Studie aus den Niederlanden [32] relevant, die die weitverbreitete Annahme infrage stellt, dass Teenager schnell vom Küssen zum penetrativen Sex übergehen. Sie zeigt stattdessen eine schrittweise Entwicklung der sexuellen Kompetenzen auf. Jugendliche, die sich die Zeit nehmen, diese Kompetenzen in jeder Phase aufzubauen, neigen weniger zu risikoreichem Verhalten.

Mit dem zunehmenden Konsens über die Notwendigkeit, die emotionalen, körperlichen und kognitiven Aspekte der sexuellen Entwicklung von Geburt an zu unterstützen, werden Forderungen nach mehr kind- und jugendzentrierten CSE-Inhalten laut. Einige Forschende [31, 33] kritisieren, dass der Fokus der CSE nach wie vor nicht auf grundlegenden Kompetenzen, Fähigkeiten und Erfahrungen liegt, die für die frühe sexuelle Entwicklung von zentraler Bedeutung sind.

### Lust und Verlangen bleiben Lücken in der CSE

Bereits 1988 wies die Wissenschaftlerin Michelle Fine auf das „Lustdefizit“ in der Sexualerziehung hin [34]. Seitdem haben zahlreiche Forschungsarbeiten den fehlenden Diskurs über das Verlangen hervorgehoben [35–37] – trotz eindeutiger Studien, die zeigen, dass junge Menschen nicht nur durch die fehlende Thematisierung von Leidenschaft in der CSE „abgeschreckt“ werden [38], sondern dass dieses Defizit sie dazu bringt, erotische Informationen in Quellen zu suchen, deren Qualität und Genauigkeit weniger sicher sind [35, 37, 39].

Das stärkste Argument für die Einbeziehung von Lust in die CSE ist unter Umständen ganz einfach: Sie ist in jedem Alter eine wichtige Triebfeder für Intimität und sexuelle Aktivität [40–42]. Mit anderen Worten: Ohne einen Diskurs über Lust wird die CSE für das Leben von Jugendlichen nicht so einen hohen Stellenwert haben. Was jedoch vielleicht noch besorgniserregender ist: Es wird eine Gelegenheit verpasst, vorhandenen Mythen und den „vorherrschenden Diskursen“ entgegenzuwirken, die „weibliche Sexualität als passiv und an der Rolle als Objekt und Opfer orientiert“ darstellen [35]. Darüber hinaus zeigen die Erkenntnisse aus dem breiteren Bereich der sexuellen und reproduktiven Gesundheit und Rechte (SRHR), dass lustbejahende Ansätze zu besseren Gesundheitsergebnissen führen können. Mindestens eine systematische Analyse, die sich mit den Auswirkungen der Einbeziehung von Lust in SRHR-Interventionen befasste, kam zu dem Ergebnis, dass sich die Resultate im Bereich der sexuellen Gesundheit verbesserten [42].

Die Offenheit gegenüber sexueller Lust als wichtigen „Bestandteil“ von CSE- und SRHR-Programmen im weiteren Sinne ist angesichts des gleichzeitigen Anstiegs des politischen und ideologischen Widerstands gegen SRHR besonders wichtig [41]. Auch bietet die Offenheit hinsichtlich sexueller Lust als Thema von CSE und SRHR neue Wertvorstellungen für die Einbeziehung sexualitätsbejahender Ansätze, die sich auf Lust als wesentlichen Bestandteil von Gesundheit und Wohlbefinden konzentrieren und eng mit der Verwirklichung von Menschenrechten verbunden sind [43–45].

### Der rechtsbasierten CSE einen Sinn verleihen

Seit vielen Jahren sprechen Befürwortende und Interessensvertretende von einer „rechtsbasierten“ umfassenden Sexualaufklärung (CSE). Sexualaufklärung ist als Recht in mehreren internationalen Menschenrechtsdokumenten verankert.^2^ Ergänzend bekräftigen UNESCO- und WHO-Standards, dass umfassende Sexualaufklärung ein zentraler Bestandteil menschenrechtsbasierter Bildung ist. Auch die Europäische Menschenrechtskonvention schützt Sexualaufklärung indirekt: Der Europäische Gerichtshof für Menschenrechte bestätigte wiederholt, dass staatliche Sexualerziehung zulässig und notwendig ist, solange sie sachlich und pluralistisch bleibt (Art. 2, 1. Zusatzprotokoll). Die UN-Nachhaltigkeitsziele (SDGs) stärken diesen Anspruch, da sexual- und reproduktive Gesundheitsinformationen explizit in den Zielen zu Gesundheit (SDG 3), Bildung (SDG 4) und Geschlechtergleichstellung (SDG 5) verankert sind. Mit der fortschreitenden Forschung in den Bereichen Menschenrechte und CSE haben Studien weitere Erkenntnisse erbracht. Insgesamt ergibt sich aus der Literatur zur Anwendung von Rechten bei CSE-Programmen Folgendes [9, 46]:

1. die Notwendigkeit, die Stimmen von Kindern und Jugendlichen in den Mittelpunkt zu stellen, und die Forderung, ihr Recht auf Beteiligung an allen Entscheidungen, die ihre Bildung, Gesundheit und ihr Wohlbefinden betreffen, zu garantieren;
2. die Beachtung der Art und Weise, wie marginalisierte Kinder und Jugendliche CSE erhalten, um ihr Recht auf Freiheit von Diskriminierung zu gewährleisten. Gleichzeitig haben wichtige Studien die Themen Rechte, Macht und Geschlecht mit einem größeren Potenzial für die Beeinflussung von Gesundheitsergebnissen verknüpft.

Zunehmend berücksichtigen Studien die Stimmen von Kindern, Jugendlichen und jungen Erwachsenen bei der Beurteilung der inhaltlichen Gestaltung und Vermittlung von CSE-Programmen. In England untersuchte eine Studie [47] die Erfahrungen von 10- und 11-jährigen Kindern mit der sexuellen Bildung, die dort seit 2020 verpflichtend ist. Die Ergebnisse konzentrieren sich speziell auf Themen im Zusammenhang mit sexuellem Kindesmissbrauch und zeigen eine breite und auch starke Unterstützung für den Lehrplan unter den Kindern, bei gleichzeitiger Betonung der Relevanz von CSE für ihr Leben. In Neuseeland und den USA zeigen Untersuchungen [38], wie wichtig die Zusammenarbeit mit Teenagern ist, um Lücken im Lehrplan zu identifizieren – insbesondere in Bezug auf Lust –, indem bewertet wird, was sie sich von der CSE wünschen. Eine in der Schweiz durchgeführte Umfrage unter mehr als 4500 jungen Erwachsenen [48] verglich ihre Informationsquellen über Sexualität während der Adoleszenz mit Indikatoren für sexuelles Verhalten. Die Ergebnisse zeigen, dass die Schülerschaft, die ihre Informationen hauptsächlich von Bekannten, aus dem Internet, aus anderen Quellen oder gar von niemandem erhielten, eher über *ungewollte *sexuelle Erfahrungen berichteten als diejenigen, die ihre Informationen in der Schule erhielten.

Die Forschung mit Kindern und Jugendlichen ist nur eine Möglichkeit, deren Recht auf Mitbestimmung hinsichtlich Entscheidungen zu fördern, welche sie selbst betreffen. Trotzdem birgt sie großes Potenzial, wenn es darum geht, sicherzustellen, dass die Lebensrealität von Kindern und Jugendlichen mit politischen Maßnahmen und Programmen, die sich auf ihre Gesundheit und ihr Wohlbefinden auswirken sollen, im Einklang steht [49].

Das Recht auf eine nichtdiskriminierende Umgebung wurde im Zusammenhang mit CSE so ausgelegt, dass sie die Vielfalt der Erfahrungen berücksichtigen muss, einschließlich der Erfahrungen von Kindern und Jugendlichen mit Beeinträchtigungen sowie Menschen mit Migrations- oder Fluchthintergrund, anderer Hautfarbe und Menschen, die sich als LGBTQIA+ identifizieren. Die Literatur zeigt die Stärken von CSE auf, weist aber auch auf bestehende Lücken und Hindernisse hin. Eine Übersichtsarbeit im europäischen Kontext [50] zeigte, dass CSE zwar das Potenzial hat, junge Menschen mit Beeinträchtigung zu unterstützen, dass aber nach wie vor eine Reihe von Hindernissen besteht, darunter der Mythos, sie seien asexuell, und die Übertragung der Informationspflicht für diese Gruppe auf Dritte. Mehrere Studien adressieren die Barrieren, die transgeschlechtliche junge Menschen bei der Beschaffung von für sie relevanten Informationen in CSE-Programmen erleben. Nach der Analyse von YouTube-Videos, die beschreiben, wie transgeschlechtliche junge Menschen über ihren eigenen Körper sprechen, plädieren Riggs und Bartholomew [51] dafür, den Fokus eher auf die „Funktionalitäten“ von Körperteilen zu legen als auf das geschlechtsspezifische Verständnis, das oft in der CSE verankert ist.

Auch die Einstellung der verantwortlichen Lehrkräfte kann bei der Validierung der Erfahrungen von Jugendlichen eine große Rolle spielen. Untersuchungen aus Australien [52] zeigen, dass die positive Einstellung der Lehrkräfte gegenüber Geschlechtervielfalt für das Wohlbefinden transgeschlechtlicher Lernender in weiterführenden Schulen entscheidend ist.

Insgesamt lenken die neuen Forschungsergebnisse die Aufmerksamkeit auf das große Potenzial inklusiver und vielfaltsbezogener Ansätze, die das Recht aller Kinder und Jugendlichen auf ein diskriminierungsfreies Leben fördern, auch wenn praktische Lösungen in der Literatur noch fehlen. Eine Studie aus Schweden [53] befasst sich mit diesem Thema und weist auf die Bedeutung „normkritischer“ Ansätze für die CSE hin. Sie liefert sehr praktische, im Rahmen der Studie beobachtete Strategien zur Gewährleistung von Inklusion, darunter die sensible Verwendung von Sprache, die Organisation und Einbeziehung „sensibler“ Inhalte, um Stigmatisierung entgegenzuwirken, sowie die Verwendung verschiedener Modalitäten zur Schaffung einer spezifischen Wissensordnung.

## Diskussion

Seit der Veröffentlichung der Standards im Jahre 2010 hat sich ein umfangreicher Forschungsbestand zu CSE entwickelt, begleitet von neuen theoretischen und praktischen Diskursen. Lange Zeit wurde CSE vor allem aus Sicht der öffentlichen Gesundheit bewertet, mit Schwerpunkt auf Ergebnissen wie sinkenden Schwangerschaftsraten, HIV und anderen sexuell übertragbaren Krankheiten. Diese Aspekte bleiben weiterhin relevant, doch zeigen neuere Forschungsansätze, dass CSE darüber hinaus eine Vielzahl von Faktoren beeinflusst, darunter die Verbesserung von Beziehungen, die Förderung sozialemotionaler Kompetenzen sowie die Verringerung geschlechtsspezifischer Gewalt und Diskriminierung.

Gleichzeitig hat sich gezeigt, dass in den kommenden Jahren weitere Forschung und Schwerpunktsetzung im Bereich CSE erforderlich sind, um die Thematik in ihrer Gänze zu erfassen. Dazu gehören die Folgenden:

- *Queering CSE:* Die Wissenschaft plädiert dafür, heteronormative Narrative infrage zu stellen, um vielfältige Familienstrukturen und Ausdrucksformen von Sexualität darzustellen.
- *Digitale Realitäten:* Junge Menschen nutzen zunehmend soziale Medien und KI-gesteuerte Plattformen, um sich über sexuelle Gesundheit zu informieren, was eine digitale Kompetenz schon ab einem frühen Alter erforderlich macht. Das kann sowohl neue Chancen wie auch Risiken zur Folge haben, die weiter erforscht werden sollten.
- *Umsetzung und Ausweitung:* Studien haben förderliche Bedingungen für eine effektive Integration von CSE identifiziert, darunter die Fortbildung von Lehrkräften, die Einbeziehung der Eltern und die Unterstützung durch die politischen Rahmenbedingungen.
- *Dekolonisation: *Erhöhung der Sensibilisierung für kultursensible CSE auch in der Forschung.
- *Oppositionsstrategien:* Der organisierte Widerstand gegen CSE hat sich verstärkt. Fachkräfte betonen, dass Debatten neu gestaltet werden müssen, um wissenschaftliche Nachweise, Werte und positive Ergebnisse hervorzuheben, anstatt Mythen zu reproduzieren.

Die bereits gewonnenen Erkenntnisse unterstreichen, wie wichtig es ist, CSE an die sich entwickelnden Fähigkeiten von Kindern anzupassen, beginnend in der frühen Kindheit, und rechtsbasierte und inklusive Ansätze zu integrieren, die vielfältige Erfahrungen und Realitäten widerspiegeln. Die frühzeitige und altersgerechte Auseinandersetzung mit Themen wie Lust, Geschlecht und Macht erhöht nicht nur die Relevanz von CSE für das Leben von Kindern und Jugendlichen, sie hinterfragt auch schädliche Narrative über die „Unschuld der Kindheit“, die ihre Handlungsfähigkeit eingrenzen. Die Ergebnisse deuten darauf hin, dass zukünftige Forschung und die Umsetzung von CSE – einschließlich der Standards – besonderes Augenmerk auf Inklusion, digitale Realitäten und Strategien zur Bekämpfung wachsender Widerstände legen sollten.

### Limitationen

Die Suchstrategie dieses Reviews war zielgerichtet und nicht vollständig umfassend; daher könnten einige relevante Studien unberücksichtigt geblieben sein – insbesondere nichtenglischsprachige europäische Literatur sowie Materialien, die in systematischen Reviews nicht zitiert wurden. Der Review legte mehr Wert auf Breite und konzeptionelle Klarheit als auf Vollständigkeit. Künftige Reviews würden von erweiterten mehrsprachigen Suchstrategien und einer systematischeren Abdeckung wissenschaftlicher Datenbanken profitieren. Ein möglicher Bestätigungsfehler („confirmation bias“) wurde durch die Anwendung vordefinierter Kriterien, vielfältige Evidenzreferenzen und iterative Analyseprozesse gemindert.

## Fazit

Die Analyse zeigt eine inhaltliche Weiterentwicklung von CSE, die evidenzbasiert in die Überarbeitung der Standards einbezogen werden sollte. Seit 2010 hat sich die Forschung zu CSE deutlich weiterentwickelt und zeigt, dass der Ansatz weit über gesundheitliche Effekte hinausgeht. Neben der Prävention von Schwangerschaften und sexuell übertragbaren Infektionen trägt CSE zur Förderung sozialemotionaler Kompetenzen, zur Stärkung von Beziehungen sowie zur Reduktion von Gewalt und Diskriminierung bei. Zukünftig sollte besonderes Augenmerk auf Inklusion, die Berücksichtigung digitaler Lebenswelten und Strategien zur Auseinandersetzung mit wachsendem Widerstand gelegt werden, um die Relevanz, Wirksamkeit und Akzeptanz von CSE im europäischen Kontext zu stärken.

### Infobox Im Fokus: Sexualität im frühen Kindesalter in Finnland

Finnische Forschende führten eine landesweite Umfrage unter mehr als 500 Fachkräften aus dem Bereich der frühkindlichen Bildung und Betreuung durch, um die Ausdrucksformen der Sexualität bei 1‑ bis 6‑Jährigen zu verstehen. Über 70 % der Befragten gaben an, dass Kinder ihre Emotionen, Wünsche und Bedürfnisse bereitwillig und häufig zeigten; 30 % beobachteten oft, dass Kinder die Privatsphäre anderer verstehen und respektieren. Die Hälfte der Fachkräfte gab an, häufig beobachtet zu haben, dass Kinder heimlich sexuelle Erkundungen vornehmen; die Mehrheit habe bei Kindern, die sich in ihrer Obhut befanden, spielerisches Verhalten beobachtet, das aus Erwachsenenperspektive als Masturbation bezeichnet werden kann. Die Ergebnisse deuten darauf hin, dass auch sehr kleine Kinder altersgerechte Informationen und Fähigkeiten erwerben müssen, die ihre allgemeine Entwicklung fördern [21].

„Die frühe sexuelle Entwicklung manifestierte sich in Neugierde gegenüber dem eigenen Körper, dem Erkunden seiner Funktionen, Eigenschaften und Merkmale. Auf der emotionalen Ebene äußerte sich diese Neugierde in reichlich vorhandenen Gefühlen der Verliebtheit und Zärtlichkeit, die offen denen gegenüber gezeigt wurden, die das Kind mochte“ [21].

## Supplementary Information

ESM1: Zusatzmaterial 1

## Footnotes

1 Der Begriff „Vergewaltigungsmythen“ bezeichnet weitverbreitete, falsche Vorstellungen über Vergewaltigung, wie zum Beispiel Opfer-Täter-Umkehr, Relativierung der Schwere der Tat, (Teil‑)Schuld der Opfer (Kleidung, Provokation, Alkoholkonsum), Vorstellungen darüber, wie ein Opfer nach der Tat handeln, sich kleiden oder fühlen soll, u. Ä.

2 Zum Beispiel geht es in der UN-Kinderrechtskonvention um den Zugang zu Gesundheits- und Informationsrechten für Kinder (Art. 13, 17, 24), die CEDAW (Convention on the Elimination of All Forms of Discrimination Against Women) verpflichtet Staaten zur Bereitstellung gesundheitlicher und reproduktiver Aufklärung für Frauen (Art. 10, 12) und der Internationale Pakt über wirtschaftliche, soziale und kulturelle Rechte (IpwskR) umfasst sexuelle und reproduktive Gesundheit als Teil des Rechts auf Gesundheit (Art. 12).

## References

[1] UNESCOUNESCO; UNAIDS; UNFPA; UNICEF; UN WOMEN; WHO (ed) (2018) International technical guidance on sexualitity education. An evidence-informed approach. https://www.unfpa.org/sites/default/files/pub-pdf/ITGSE.pdf. Accessed 2.9.2024

[2] BZgA W (2010) Standards for Sexuality Education in Europe. https://whocc.bioeg.de/fileadmin/user_upload/BZgA_Standards_English.pdf. Accessed 20.9.2024

[3] Anonymous (2024) Whose Hands on our Education? In:ALiGN Advancing Learning and Innovation on Gender Norms. https://www.alignplatform.org/sites/default/files/2024-09/education-backlash-full-report.pdf. Accessed 24.11.2025

[4] Montgomery PK, Wendy (2018) Review of the evidence on sexuality education: report to form the update of the UNESCO International technical guidance on sexuality education. In:UNESCOhttps://unesdoc.unesco.org/ark:/48223/pf0000264649. Zugegriffen: 30.06.2024

[5] Wisbaum W (2022) Evidence gaps and research needs in comprehensive sexuality. https://unesdoc.unesco.org/ark:/48223/pf0000380513?posInSet=1=3963cf2f-8e78-4d90-a61c-5e17107002fe. Accessed 10.3.2024

[6] UNFPA B Overview of comprehensive sexuality education status in Georgia, Kyrgyzstan, the Republic of Moldova and Tajikistan. In:https://eeca.unfpa.org/sites/default/files/pub-pdf/2024-08/SERAT_4%20country_Summary_V4.pdf. Zugegriffen: 10.02.2025

[7] Kapella OB L (2017) Training matters: A framework for core competencies of sexuality educators. https://whocc.bioeg.de/fileadmin/user_upload/BZgA_TrainingMattersFramework_EN.pdf. Accessed 25.7.2025

[8] Goldfarb ES, Lieberman LD (2021) Three Decades of Research: The Case for Comprehensive Sex Education. J Adolesc Health 68:13–27. 10.1016/j.jadohealth.2020.07.036 33059958 10.1016/j.jadohealth.2020.07.036

[9] Haberland N, Rogow D (2015) Sexuality education: emerging trends in evidence and practice. J Adolesc Health 56:S15–S21. 10.1016/j.jadohealth.2014.08.013 25528976 10.1016/j.jadohealth.2014.08.013

[10] Kantor LM, Lindberg L (2020) Pleasure and Sex Education: The Need for Broadening Both Content and Measurement. Am J Public Health 110:145–148. 10.2105/ajph.2019.305320 31855482 10.2105/AJPH.2019.305320 PMC6951361

[11] Ketting E, Friele M, Michielsen K (2016) Evaluation of holistic sexuality education: A European expert group consensus agreement. Eur J Contracept Reprod Health Care 21:68–80. 10.3109/13625187.2015.1050715 26024010 10.3109/13625187.2015.1050715

[12] K M, O I (2022) Comprehensive sexuality education: why is it important? https://www.europarl.europa.eu/RegData/etudes/STUD/2022/719998/IPOL_STU(2022)719998_EN.pdf. Accessed 4.12.2025

[13] Gayles J, Yahner M, Barker KM et al (2023) Balancing Quality, Intensity and Scalability: Results of a Multi-level Sexual and Reproductive Health Intervention for Very Young Adolescents in Kinshasa. J Adolesc Health 73:S33–S42. 10.1016/j.jadohealth.2023.02.001 37330819 10.1016/j.jadohealth.2023.02.001

[14] Woog VKgA (2017) The Sexual and Reproductive Health Needs of Very Young Adolescents In Developing Countries. https://www.guttmacher.org/fact-sheet/srh-needs-very-young-adolescents-in-developing-countries. Accessed 25.9.2024

[15] Igras SM, Macieira M, Murphy E, Lundgren R (2014) Investing in very young adolescents’ sexual and reproductive health. Glob Public Health 9:555–569. 10.1080/17441692.2014.908230 24824757 10.1080/17441692.2014.908230 PMC4066908

[16] Anonymous (2020) Executive Summary. Very young adolescent sexual and reproductive health landscape analysis. https://resourcecentre.savethechildren.net/pdf/vya_landscape_final.pdf. Accessed 21.8.2024

[17] Anonymous (2024) Building strong foundations. What is foundational education for health and well-being? https://unesdoc.unesco.org/ark:/48223/pf0000389751?posInSet=1&queryId=2802c2d9-c9fe-4f10-89a9-23f4c7d47cfc. Accessed 25.9.2024

[18] Robinson D, MacLaughlin V, Poole J (2019) Sexual health education outcomes within Canada’s elementary health education curricula: A summary and analysis. The Canadian Journal of Human Sexuality 28:1–14. 10.3138/cjhs.2018-0036

[19] Venketsamy T, Kinear J (2020) Strengthening comprehensive sexuality education in the curriculum for the early grades. South African Journal of Childhood Education 10(1):820. 10.4102/sajce.v10i1.820

[20] Cacciatore R, Öhrmark L, Kontio J et al (2024) What do 3–6-year-old children in Finland know about sexuality? A child interview study in early education. Sex Education 24:291–310. 10.1080/14681811.2023.2188182

[21] Cacciatore RS, Ingman-Friberg SM, Lainiala LP, Apter DL (2020) Verbal and Behavioral Expressions of Child Sexuality Among 1‑6-Year-Olds as Observed by Daycare Professionals in Finland. Arch Sex Behav 49:2725–2734. 10.1007/s10508-020-01694-y 32300911 10.1007/s10508-020-01694-y

[22] Anonymous SEL in the School. A systematic approach integrates SEL across all key settings where students live and learn. In:Collaborative for Academic, Social and Emotional Learning, Chicago https://casel.org/systemic-implementation/sel-in-the-school/. Zugegriffen: 04.12.2025

[23] Fraguas D, Díaz-Caneja CM, Ayora M et al (2021) Assessment of School Anti-Bullying Interventions: A Meta-analysis of Randomized Clinical Trials. JAMA Pediatr 175:44–55. 10.1001/jamapediatrics.2020.3541 33136156 10.1001/jamapediatrics.2020.3541 PMC7607493

[24] Gaffney H, Farrington D, Ttofi M (2019) Examining the Effectiveness of School-Bullying Intervention Programs Globally: a Meta-analysis. International Journal of Bullying Prevention. 10.1007/s42380-019-0007-4

[25] Anonymous (2022) What works to prevent online violence against children? Executive Summary. https://iris.who.int/server/api/core/bitstreams/abd8db20-9394-4f4f-9e82-b73933fc3a31/content. Accessed 25.9.2024

[26] Trikic ZA A (2023) Supporting Families for Gender-Transformative Parenting. https://www.unicef.org/media/134441/file/Gender_Transformative_Parenting_Resource_Modules.pdf. Accessed 25.9.2024

[27] De La Rue L, Polanin J, Espelage D, Pigott T (2014) School-Based Interventions to Reduce Dating and Sexual Violence: A Systematic Review. Campbell Systematic Reviews 10:1–110. 10.4073/csr.2014.7

[28] Cherewick M, Lebu S, Su C, Richards L, Njau P, Dahl R (2021) Promoting gender equity in very young adolescents: targeting a window of opportunity for social emotional learning and identity development. BMC Public Health. 10.1186/s12889-021-12278-3 34923962 10.1186/s12889-021-12278-3 PMC8684613

[29] Durlak J, Weissberg R, Dymnicki A, Taylor RD, Schellinger K (2011) Enhancing students’ social and emotional development promotes success in school: Results of a meta-analysis. Child Development 82:474–501 10.1111/j.1467-8624.2010.01564.x 21291449

[30] Kågesten A, van Reeuwijk M (2021) Healthy sexuality development in adolescence: proposing a competency-based framework to inform programmes and research. Sex Reprod Health Matters 29:1996116. 10.1080/26410397.2021.1996116 34937528 10.1080/26410397.2021.1996116 PMC8725766

[31] Cacciatore R, Korteniemi-Poikela E, Kaltiala R (2019) The Steps of Sexuality—A Developmental, Emotion-Focused, Child-Centered Model of Sexual Development and Sexuality Education from Birth to Adulthood. International Journal of Sexual Health 31:1–20. 10.1080/19317611.2019.1645783

[32] van der Doef S, Reinders J (2018) Stepwise sexual development of adolescents: the Dutch approach to sexuality education. Nat Rev Urol 15:133–134. 10.1038/nrurol.2018.3 29384522 10.1038/nrurol.2018.3

[33] Fortenberry JD (2013) Puberty and adolescent sexuality. Horm Behav 64:280–287. 10.1016/j.yhbeh.2013.03.007 23998672 10.1016/j.yhbeh.2013.03.007 PMC3761219

[34] Fine M (1988) Sexuality, Schooling, and Adolescent Females: The Missing Discourse of Desire. Harvard Educational Review 58:29–54. 10.17763/haer.58.1.u0468k1v2n2n8242

[35] Garland-Levett S, Allen L (2018) The Fertile, Thorny, and Enduring Role of Desire and Pleasure in Sexuality Education. In: Lamb S, Gilbert J (eds) The Cambridge Handbook of Sexual Development: Childhood and Adolescence. Cambridge University Press, Cambridge, pp 521–536

[36] Landi N (2017) ‘Pleasure Is Not in the Science Programme!’: When Anthropology Engages with Sex Education for Teenagers. https://anthropologymatters.com/index.php/anth_matters/article/view/479. Accessed 4.12.2025

[37] Singh A, Both R, Philpott A (2021) ’I tell them that sex is sweet at the right time’ - A qualitative review of ’pleasure gaps and opportunities’ in sexuality education programmes in Ghana and Kenya. Glob Public Health 16:788–800. 10.1080/17441692.2020.1809691 32816645 10.1080/17441692.2020.1809691

[38] Allen L, Carmody M (2012) ‘Pleasure Has No Passport’: Re-visiting the Potential of Pleasure in Sexuality Education. Sex Education 12:455–468. 10.1080/14681811.2012.677208

[39] Nahar P, Reeuwijk M, Reis R (2013) Contextualising sexual harassment of adolescent girls in Bangladesh. Reproductive health matters 21:78–86. https://doi.org/10.1016/S0968–8080(13)41696‑8 23684190 10.1016/S0968-8080(13)41696-8

[40] Ford J, Vargas E, Finotelli Í Jr et al (2019) Why Pleasure Matters: Its Global Relevance for Sexual Health, Sexual Rights and Wellbeing. International Journal of Sexual Health 31:1–14. 10.1080/19317611.2019.1654587

[41] Philpott A, Larsson G, Singh A, Zaneva M, Gonsalves L (2021) How to Navigate a Blindspot: Pleasure in Sexual and Reproductive Health and Rights Programming and Research. Int J Sex Health 33:587–601. 10.1080/19317611.2021.1965690 38595783 10.1080/19317611.2021.1965690 PMC10903683

[42] Zaneva M, Philpott A, Singh A, Larsson G, Gonsalves L (2022) What is the added value of incorporating pleasure in sexual health interventions? A systematic review and meta-analysis. PLoS One 17:e0261034. 10.1371/journal.pone.0261034 35148319 10.1371/journal.pone.0261034 PMC8836333

[43] Castellanos-Usigli A, Braeken-van Schaik D (2019) The Pleasuremeter: exploring the links between sexual health, sexual rights and sexual pleasure in sexual history-taking, SRHR counselling and education. Sex Reprod Health Matters 27:1–3. 10.1080/26410397.2019.1690334 31752632 10.1080/26410397.2019.1690334 PMC7887986

[44] Braeken DCU, A (2018) Sexual Pleasure. The forgotten link in sexual and reproductive health and rights. Training Toolkit. In:Global Advisory Board (GAB) for Sexual Health and Wellbeing. https://www.gab-shw.org/media/1024/gab_sexualpleasuretrainingtoolkit_final_webversion_withhyperlinks_updateapril2019.pdf. Accessed 1.10.2024

[45] Gruskin S, Yadav V, Castellanos-Usigli A, Khizanishvili G, Kismödi E (2019) Sexual health, sexual rights and sexual pleasure: meaningfully engaging the perfect triangle. Sex Reprod Health Matters 27:1593787. 10.1080/26410397.2019.1593787 31533569 10.1080/26410397.2019.1593787 PMC7887957

[46] Haberland NA (2015) The case for addressing gender and power in sexuality and HIV education: a comprehensive review of evaluation studies. Int Perspect Sex Reprod Health 41:31–42. 10.1363/4103115 25856235 10.1363/4103115

[47] Farrelly N, Barter C, Stanley N (2022) Ready for Relationships Education? Primary school children’s responses to a Healthy Relationships programme in England. Sex Education 23:1–17. 10.1080/14681811.2022.2052834

[48] Barrense-Dias Y, Akre C, Surís JC et al (2020) Does the Primary Resource of Sex Education Matter? A Swiss National Study. J Sex Res 57:166–176. 10.1080/00224499.2019.1626331 31215800 10.1080/00224499.2019.1626331

[49] Neary A (2023) Intersections of age and agency as trans and gender diverse children navigate primary school: listening to children in (re)considering the potential of sexuality education. Sex Education 24:1–15. 10.1080/14681811.2023.2238634

[50] Michielsen K, Brockschmidt L (2021) Barriers to sexuality education for children and young people with disabilities in the WHO European region: a scoping review. Sex Education 21:1–19. 10.1080/14681811.2020.1851181

[51] Riggs D, Bartholomaeus B (2017) Transgender young people’s narratives of intimacy and sexual health: implications for sexuality education. Sex Education 18:1–15. 10.1080/14681811.2017.135529931275062

[52] Ullman J (2016) Teacher positivity towards gender diversity: exploring relationships and school outcomes for transgender and gender-diverse students. Sex Education 17:1–14. 10.1080/14681811.2016.1273104

[53] Bengtsson J, Bolander E (2019) Strategies for inclusion and equality – ‘norm-critical’ sex education in Sweden. Sex Education 20:1–16. https://doi.org/10.1080/14681811.2019.1634042

