# Supplementary material for: International findings on sex education: a 15-year review (English version)
Source: Bundesgesundheitsblatt Gesundheitsforschung Gesundheitsschutz. 2026 Mar 24;69(4):465–72. [Article in German] doi: 10.1007/s00103-026-04219-5 (PMC13043613; doi:10.1007/s00103-026-04219-5)
Supplement: Supplementary file 1 — ESM1: Zusatzmaterial 1 [file 103_2026_4219_MOESM1_ESM.pdf]

This is an English translation of the article: ‚Internationale Erkenntnisse zur Sexualaufklärung: 15 Jahre im Rückblick' (Bundesgesundheitsblatt April 2026). The responsibility for the translation lies solely with the authors. Please note that only the original German-language article can be cited.

International findings on sex education: a 15-year review

Katherine Watson[1] , Volker Schmidt-Cox[2] , Johanna Marquardt[3]

[1] Freelance human rights consultant, London, United Kingdom

[2] Federal Institute for Public Health (BIÖG)/Sex Education Department, Cologne, Germany

[3] Federal Institute for Public Health (BIÖG)/WHO Collaborating Centre for Sexual and Reproductive Health, Cologne, Germany

Correspondence address:

Johanna Marquardt

Federal Institute for Public Health (BIÖG)

Maarweg 149–161

50825 Cologne

johanna.marquardt@bioeg.de

Abstract

Introduction: Comprehensive Sexuality Education (CSE) is central to the healthy development of young people, promoting healthy relationships and gender equality. Since the publication of the WHO Standards in 2010, the evidence base has expanded considerably and new discourses have gained importance. The aim of this scoping review is to provide a systematic overview of the evidence on school-based CSE published since 2010 and to identify emerging thematic developments.

Methods: As part of a scoping review, structured literature searches were carried out in PubMed, Google Scholar, and the journal Sex Education. Search terms combined variations of “comprehensive sexuality education,” “sex education,” “school-based,” “adolescence,” “sexual development,” and “Europe,” as well as terms related to child and adolescent sexual development. The search period covered January 1, 2010 to February 25, 2025. To strengthen European representation, additional references were solicited from members of the expert group. This was followed by screening, evidence extraction, and validation of the synthesis.

Results: The evidence demonstrates positive effects of CSE on social-emotional learning, healthy relationships, gender equality, and violence prevention. Increasing emphasis is placed on rights-based, inclusive, and diversity-sensitive approaches, as well as on addressing digital realities. At the same time, growing societal resistance is evident.

Discussion: The analysis highlights a substantive evolution of CSE that should be considered in the evidence-based revision of the Standards to ensure their relevance and applicability within the European context.

Key words: sexuality education, sexual development, children, adolescents, young people

Introduction

Comprehensive sexuality education (CSE) is a cornerstone of child and adolescent development, laying the foundation for health and well-being, lifelong healthy relationships, and gender equality. CSE is recognised globally as an important strategy for promoting positive outcomes in sexual development and is defined in the International Technical Guidance on Sexuality Education (ITGSE) as follows:

> "...a curriculum-based process of teaching and learning about the cognitive, emotional, physical and social aspects of sexuality. It aims to equip children and young people with knowledge, skills, attitudes and values that will empower them to: realise their health, well-being and dignity; develop respectful social and sexual relationships; consider how their choices affect their own well-being and that of others; and, understand and ensure the protection of their rights throughout their lives." [1]

Since the 1960s, remarkable progress has been made in Europe in implementing CSE programmes in schools, although progress has been uneven. The scope, quality and implementation of school programmes vary greatly, both nationally and among the 53 countries that belong to the World Health Organization Regional Office for Europe (WHO Europe) [2] . In response, the WHO Collaborating Centre for Sexual and Reproductive Health, based at the Federal Institute of Public Health (WHO CC of the BIÖG), in collaboration with the European Expert Group on Sexuality Education (hereinafter referred to as the "Expert Group") and WHO Europe, developed and published the "Standards for Sexuality Education in Europe: A Framework for Policy Makers, Educational and Health Authorities and specialists" (hereinafter referred to as the "Standards"). The Standards have since been translated into 15 languages and are being applied throughout Europe.

In the years since the Standards were published, the international scientific basis for CSE has grown significantly. Today, the available evidence shows more clearly than ever that CSE has the potential to positively influence the health and well-being of children and young people in many ways and to contribute to their social and emotional development. At the same time that the evidence base for CSE has grown, so too has resistance from both governmental and non-governmental actors [3] . Professionals in education, science, advocacy and social work across Europe face strong opposition, ranging from online campaigns to undermine national CSE programmes, often with targeted misrepresentations, to death threats against individuals.

In light of current global challenges, the WHO CC of the BIÖG and the expert group recognised the need to revise the Standards to ensure that they remain a credible, comprehensive and authoritative reference for CSE. As a starting point, the WHO CC of the BIÖG commissioned a scoping review covering the period from the publication of the Standards (2010) to February 2025, which was further refined and validated by an expert group. The research questions are: What new evidence on school-based sexuality education has been gained since 2010, and what thematic priorities or emerging topics can be identified in the current literature?

Methods

Step 1: Defining the scope. The objectives, scope and priority topics of the review were specified at the outset in consultation with the expert group. In this step, the research questions were formulated as follows: 1) What new scientific evidence on school-based CSE research have been gained since the publication of the Standards in 2010? 2) What thematic priorities or emerging topics can be identified in the current literature?

Step 2: Literature search. Targeted searches were conducted in the PubMed database and via Google Scholar. Another important source in the field of CSE was the renowned journal Sex Education. The search terms used combined variants of: "comprehensive sexuality education", "sex education", "school-based", "adolescence", "sexual development" and "Europe". In addition, Google Scholar searches were conducted on the connection between child and adolescent sexual development and CSE, using the terms: "child", "adolescent", "sexual development", "sex education" and "sexuality education". The study period for all searches was set from 1 January 2010 to 25 February 2025.

Step 3: Supplementing the literature. The literature search yielded fewer studies from Europe than in the globally published literature. In order to increase regional representation, a survey was conducted among the members of the expert group, requesting additional references from European contexts. This resulted in 32 additional references in English, French, German, Spanish and Portuguese. At the same time, reference lists from the systematic reviews identified in step 2 were screened. Steps 2 and 3 identified a total of 242 studies.

Step 4: Screening and recording of evidence. Studies from all references were screened and recorded if they met the following inclusion criteria:

• dealt with school-based CSE programmes,
• examined CSE outcomes, implementation or innovation, or established a link between child/adolescent sexual development and CSE, and
• were peer-reviewed studies, systematic reviews or high-quality grey literature.

Studies that addressed extracurricular or hybrid CSE programmes or had no relation to CSE or the sexual development of children and adolescents were excluded. Ninety references met the inclusion criteria and were recorded in a review matrix documenting study type, context and relevance. Approximately 53 of these references are directly cited in this review; the remainder were used to develop the thematic framework.

Step 5: Synthesis and validation by experts. The results were synthesised in Excel using narrative, thematic analysis. The themes were derived inductively, further developed through discussions between the authors of this article, and validated by the expert group. A face-to-face meeting of the expert group in August 2024 served as a formal consultation and contributed to further refinement and validation of the emerging themes.

Results

CSE has an effect on multiple health indicators

In the past, the effectiveness of CSE was primarily measured in studies using public health indicators – particularly those relating to sexual and reproductive health. These included reductions in teenage pregnancy rates and HIV and other sexually transmitted infections (STIs) [4, 5] . Accordingly, CSE content was well suited to reducing risks. In many contexts in Western Europe and worldwide, CSE was understood as a state response to new phenomena in public health and society, including the emergence and spread of HIV, the availability of the contraceptive pill and the decriminalisation of abortion.

However, over the past 10 to 15 years, calls to expand research have grown louder [4, 5, 8-10] . In 2016, the expert group pointed out that "the current literature demonstrates that evaluation criteria predominantly focus on the public health impact " – but often at the expense of more positive indicators for sexuality and for the development of children and adolescents [11] .

In response to the narrow evidence and calls for recognition of the broader potential of CSE, the scientific community began to expand research over the past 10 years [4, 8, 12] . In their search for studies that measured CSE outcomes other than solely the reduction of STIs or pregnancies, Goldfarb and Leiberman [8] identified 48 articles in their systematic review, 39 of which were from the United States. They grouped these studies into several categories based on the measurements used:

- appreciation of diversity,
- Prevention of violence in relationships and dating,
- healthy relationships,
- Prevention of child abuse, and
- social-emotional learning and digital literacy [8] .

In addition to expanding measurement methods, some studies have also attempted to move away from a "risk approach" and instead focus on the positive effects of CSE on young people's lives. For example, in 2022, UNESCO commissioned a study, the results of which will be published shortly, to explore the potential of school-based CSE to improve learners' relationships with their parents; young people and their partners were surveyed in six countries.

Experts also called for more holistic measurement methods that move away from randomised controlled trials (RCTs) as the gold standard and instead focus more on the importance of qualitative evidence and the perspectives of young people:

> *"Mixed methods research — involving RCTs and associated qualitative Review of the Evidence on Sexuality Education research in the context of the trial — are vital for understanding the many facets of effectiveness as they apply in educational settings and in the lives of young people. Likewise, the word 'effectiveness' can be understood in different ways, depending on the goals of a particular programme or effort. For instance, in educational settings, there are overarching goals that go far beyond measuring a single programme over a short period of time (e.g., one school year)."* [4]

Critical developmental stages offer opportunities for CSE

Since 2010, research has shown a growing interest in how sexual and reproductive health and rights (SRHR) and, in particular, CSE programmes for younger age groups should be examined in a

differentiated manner. Various studies have addressed the overlapping categories of "very young adolescents" aged 10 to 14 [13-16] and "primary school children" aged 5 to 12 [17-19] . Few studies have focused on preschool or kindergarten-aged children, although research in Finland with preschool-aged children (1–6 years) shows that sexuality is part of the lifelong development process from an early age (see Info Box 1) [20, 21] .

To emphasise the importance of developmentally appropriate CSE for children and young people of all ages and to illustrate the potential of each developmental period for specific outcomes, research draws on studies of child and adolescent development. During their preschool and primary school years, children undergo a series of cognitive, neurological, social, emotional and physical developments, from an increased capacity for abstract thinking to a better understanding of stereotypes, gender roles and self-identification [17, 20] . All of this contributes to sexual development. These rapidly successive stages of development offer opportunities to improve health and well-being both in childhood and adolescence and throughout life, including in relation to:

- promoting skills that support lifelong learning;
- providing knowledge and teaching skills to prevent abuse and deal with (sexualised) violence;
- reducing bullying in schools and on the internet;
- reducing stress levels and increasing self-efficacy;
- improving coping skills and other social and emotional skills;
- improving sexual and reproductive health later in life;
- promoting gender equality;
- the contribution to active social engagement [8, 17, 22-25].

Particular attention was given in the literature to the potential of CSE programmes to contribute to the formation of equitable gender norms, which begin to develop as early as age 5 [17, 26] . There is evidence that the early school years are the best time to introduce sexuality-related topics and that addressing gender and power dynamics in CSE leads to better outcomes on a range of other health and wellbeing indicators [8, 9].

> "*This review suggests that not only are younger children able to discuss sexuality-related issues but that the early grades may, in fact, be the best time to introduce topics related to sexual orientation, gender identity and expression, gender equality, and social justice related to the*

LGBTQ community before hetero- and cisnormative values and assumptions become more deeply ingrained and less mutable. Children learn gender role attitudes at an early age from observing the people in their families." [8]

Research indicates that CSE in early adolescence can lead to further positive outcomes, including lower acceptance of rape myths[1] later in life [27], a decline in sexual violence [8, 19] , the development and maintenance of healthy relationships throughout life (UNESCO, forthcoming), prosocial behavior such as kindness, willingness to share and empathy, and associated higher educational attainment [28, 29] , as well as sexual well-being [30] .

CSE should be tailored to the development of children and adolescents

The literature on child and adolescent development promotes recognition of the need to move away from an understanding of sexuality that focuses exclusively on coitus and to develop an understanding of sexuality as something distinct at each stage of life. Cacciatore and colleagues [31] have formulated "stages of sexuality" that provide a framework for sexual development from birth to adolescence. The stages range from "I am wonderful" (discovery of one's own body and interest in the differences between the sexes: infancy and early childhood) to "maturity for love" (readiness for the first sexual experience: middle to late adolescence). Although the authors believe that development does not necessarily proceed in a linear fashion through the individual stages, the "goals" of each stage must be achieved in order to move on to the next. It is important that children and adolescents receive information about each stage in advance.

Although empirical research on sexual development and CSE is limited, some studies from the European context show that basic sexuality-related skills developed in infancy are further developed in childhood and adolescence. A study with Finnish educators found that the emotional and physical foundations of healthy sexuality are already laid at the age of one [21]. A study from the Netherlands [32] is relevant to the sexual development of adolescents, challenging the widespread assumption that teenagers quickly progress from kissing to penetrative sex. Instead, it shows a gradual development of sexual skills. Adolescents who take the time to build these skills at each stage are less prone to risky behavior.

---

[1] The term "rape myths" refers to widespread misconceptions about rape, such as victim-blaming, downplaying the severity of the crime, (partial) blame of the victim (clothing, provocation, alcohol consumption), ideas about how a victim should act, dress or feel after the crime, etc.

Info box 1

In focus: sexuality in early childhood in Finland
Finnish researchers conducted a nationwide survey of more than 500 early childhood education and care professionals to understand the expressions of sexuality in 1- to 6-year-olds. Over 70% of respondents said that children willingly and frequently showed their emotions, desires and needs; 30% often observed that children understood and respected the privacy of others. Half of the professionals said they had often observed children secretly engaging in sexual exploration; the majority had observed playful behaviour in children in their care that could be described as masturbation from an adult perspective. The findings suggest that even very young children need to acquire age-appropriate information and skills that promote their overall development [21].

"Early sexual development manifested as curiosity about one's own body, exploring its functions, traits, and attributes, while on an emotional level it manifested as abundant feelings of infatuation and tenderness, shown openly toward those—peers and adults alike—whom the child cares for." [21]

With growing consensus on the need to support the emotional, physical and cognitive aspects of sexual development from birth, there are calls for more child- and youth-centred CSE content. Some researchers [31, 33] criticise that CSE still does not focus on the basic skills, abilities and experiences that are central to early sexual development.

Pleasure and desire remain gaps in CSE

As early as 1988, researcher Michelle Fine pointed out the "desire deficit" in sexuality education [34]. Since then, numerous research papers have highlighted the lack of discourse on desire [35-37] – despite clear studies showing that young people are not only "put off" by the lack of discussion of passion in CSE [38] , but that this deficit leads them to seek erotic information in sources whose quality and accuracy are less certain [35, 37, 39].

The strongest argument for including pleasure in CSE may be quite simple: it is an important driver of intimacy and sexual activity at any age [40-42]. In other words, without a discourse on pleasure, CSE will not be as relevant to young people's lives. What is perhaps even more worrying, however, is that it misses an opportunity to counter existing myths and " dominant discourses of female sexuality as passive, objectified and victimised" [35]. Furthermore, evidence from the broader field of SRHR shows that pleasure-affirming approaches can lead to better health outcomes. At least one systematic review examining the impact of incorporating pleasure into SRHR interventions concluded that sexual health outcomes improved [42].

Openness to sexual pleasure as an important "component" of CSE and SRHR programmes in the broader sense is particularly important given the simultaneous rise in political and ideological resistance to SRHR [41]. Openness about sexual pleasure as a topic of CSE and SRHR also offers new values for the inclusion of sexuality-affirming approaches that focus on pleasure as an essential component of health and well-being and are closely linked to the realisation of human rights [43-45].

Giving meaning to rights-based CSE

For many years, advocates and interest groups have been talking about "rights-based" CSE. Sexuality education is enshrined as a right in several international human rights documents[2] . In addition, UNESCO and WHO Standards affirm that CSE is a central component of human rights-based education. The European Convention on Human Rights also indirectly protects sexuality education: the European Court of Human Rights has repeatedly confirmed that state sexuality education is permissible and necessary as long as it remains factual and pluralistic (Art. 2, 1st Additional Protocol). The UN Sustainable Development Goals (SDGs) reinforce this claim, as sexual and reproductive health information is explicitly anchored in the goals on health (SDG 3), education (SDG 4) and gender equality (SDG 5). With ongoing research in the areas of human rights and CSE, studies have yielded further insights. Overall, the literature on the application of rights in CSE programmes has revealed the following [9, 46]:

1) the need to focus on the voices of children and young people and the demand to guarantee their right to participate in all decisions affecting their education, health and well-being; and

2) consideration of how marginalised children and young people receive CSE in order to ensure their right to freedom from discrimination.

At the same time, important studies have linked the issues of rights, power and gender with greater potential to influence health outcomes.

Increasingly, studies are taking into account the voices of children, adolescents and young adults when assessing the content design and delivery of CSE programmes. In England, one study [47] examined the

[2] For example, the UN Convention on the Rights of the Child deals with children's access to health and information rights (Articles 13, 17, 24), the CEDAW (Convention on the Elimination of All Forms of Discrimination Against Women) obliges states to provide health and reproductive education for women (Articles 10, 12), and the International Covenant on Economic, Social and Cultural Rights (ICESCR) includes sexual and reproductive health as part of the right to health (Art. 12).

experiences of 10- and 11-year-old children with sexuality education, which has been compulsory there since 2020. The findings focus specifically on issues related to child sexual abuse and show broad and strong support for the curriculum among children, while emphasising the relevance of CSE to their lives. In New Zealand and the US, research [38] shows how important it is to work with teenagers to identify gaps in the curriculum – particularly in relation to pleasure – by assessing what they want from CSE. A survey of more than 4,500 young adults conducted in Switzerland [48] compared their sources of information about sexuality during adolescence with indicators of sexual behavior. The results showed that students who obtained their information mainly from acquaintances, the internet, other sources or even from no one at all were more likely to report *unwanted* sexual experiences than those who obtained their information at school.

Research with children and young people is only one way of promoting their right to have a say in decisions that affect them. Nevertheless, it has great potential when it comes to ensuring that the realities of children's and young people's lives are reflected in policies and programmes that are intended to affect their health and well-being [49].

The right to a non-discriminatory environment has been interpreted in the context of CSE as requiring that it must take into account the diversity of experiences, including those of children and young people with disabilities, as well as people with a migrant or refugee background, people of color and people who identify as LGBTQIA+. The literature highlights the strengths of CSE, but also points to existing gaps and barriers. A review in the European context [50] showed that while CSE has the potential to support young people with disabilities, a number of barriers remain, including the myth that they are asexual and the transfer of the duty to provide information for this group to third parties. Several studies address the barriers that transgender young people experience in obtaining information relevant to them in CSE programmes. After analysing YouTube videos describing how transgender young people talk about their own bodies, Riggs and Bartholomew [51] argue for focusing on the "functionalities" of body parts rather than the gender-specific understanding that is often embedded in CSE.

The attitude of the teachers responsible can also play a major role in validating young people's experiences. Research from Australia [52] shows that teachers' positive attitudes towards gender diversity are crucial to the well-being of transgender learners in secondary schools.

Overall, the new research findings draw attention to the great potential of inclusive and diversity-related approaches that promote the right of all children and young people to a life free of discrimination, even if practical solutions are still lacking in the literature. A study from Sweden [53]

addresses this issue and points to the importance of "norm-critical" approaches to CSE. It provides very practical strategies for ensuring inclusion that were observed in the study, including the sensitive use of language, the organisation and inclusion of "sensitive" content to counter stigmatisation, and the use of different modalities to create a specific knowledge order.

Discussion

Since the publication of the Standards in 2010, a substantial body of research on CSE has developed, accompanied by new theoretical and practical discourses. For a long time, CSE was primarily evaluated from a public health perspective, with a focus on outcomes such as declining pregnancy rates, HIV and other sexually transmitted diseases. These aspects remain relevant, but recent research approaches show that CSE also influences a variety of factors, including improving relationships, promoting social-emotional skills, and reducing gender-based violence and discrimination.

At the same time, it has become clear that further research and focus within CSE will be needed in the coming years. This includes promoting greater understanding of the following topics:

- *Queering CSE:* CSE has the potential to promote inclusivity and diversity in the way that it challenges heteronormative narratives and represents diverse family structures and expressions of sexuality.
- *Digital realities:* Young people are increasingly using social media and AI-driven platforms to learn about sexual health; CSE has the potential to enhance digital literacy from an early age.
- *Implementation and expansion:* Studies have identified conditions conducive to the effective implementation of CSE, including teacher training, parental involvement and support from the political framework, but more is needed given the diversity of education systems and socio-cultural contexts within which CSE is provided.
- Decolonisation: Raising awareness of culturally sensitive CSE in research.
- *Opposition strategies:* Organised resistance to CSE has intensified; experts emphasise that debates need to be reframed to highlight scientific evidence, values and positive outcomes rather than reproducing myths.

The findings already obtained underscore the importance of adapting CSE to children's developing capacities, starting in early childhood, and of integrating rights-based and inclusive approaches that reflect diverse experiences and realities. e and age-appropriate engagement with topics such as pleasure, gender and power not only increase the relevance of CSE to the lives of children and young people, it also challenges harmful narratives about the 'innocence of childhood' that limit their agency. The findings suggest that future research and implementation of CSE – including standards – should pay particular attention to inclusion, digital realities and strategies to combat growing resistance.

Limitations. The search strategy for this review was targeted and not comprehensive; therefore, some relevant studies may have been overlooked – in particular, non-English-language European literature and materials not cited in systematic reviews. The review emphasised breadth and conceptual clarity over completeness. Future reviews would benefit from expanded multilingual search strategies and more systematic coverage of scientific databases. Potential confirmation bias was mitigated by the use of predefined criteria, diverse evidence references, and iterative analysis processes.

Conclusion

The analysis shows a further development of CSE in terms of content, which should be incorporated into the revision of the Standards in an evidence-based manner. Since 2010, research on CSE has developed significantly and shows that the outcomes spread far beyond health. In addition to preventing pregnancies and sexually transmitted infections, CSE contributes to the promotion of social-emotional skills, the strengthening of relationships and the reduction of violence and discrimination. In future, particular attention should be paid to inclusion, consideration of digital environments and strategies for dealing with growing resistance in order to strengthen the relevance, effectiveness and acceptance of CSE in the European context.

Compliance with ethical guidelines

Conflict of interest

K. Watson, J. Marquardt and V. Schmidt-Cox declare that there is no conflict of interest.

The authors did not conduct any studies on humans or animals for this article. The ethical guidelines specified in each case apply to the studies listed.

References

1. UNESCO (2018) International technical guidance on sexuality education. An evidence-informed approach. In: UNESCO; UNAIDS; UNFPA; UNICEF; UN WOMEN; WHO https://www.unfpa.org/sites/default/files/pub-pdf/ITGSE.pdf. 02.09.2024
2. BZgA W (2010) Standards for Sexuality Education in Europe. In: BZgA, Colognehttps://whocc.bioeg.de/fileadmin/user_upload/BZgA_Standards_English.pdf. Accessed: 20.09.2024
3. Anonymous (2024) Whose Hands on our Education? In: ALiGN Advancing Learning and Innovation on Gender Normshttps://www.alignplatform.org/sites/default/files/2024-09/education-backlash-full-report.pdf. Accessed: 24 November 2025
4. Montgomery PK, Wendy (2018) Review of the evidence on sexuality education: report to form the update of the UNESCO International technical guidance on sexuality education. In: UNESCO https://unesdoc.unesco.org/ark:/48223/pf0000264649 Accessed: 30 June 2024
5. Wisbaum W (2022) Evidence gaps and research needs in comprehensive sexuality. Technical brief. In: https://unesdoc.unesco.org/ark:/48223/pf0000380513?posInSet=1&queryId=3963cf2f-8e78-4d90-a61c-5e17107002fe. Accessed: 10 March 2024
6. UNFPA B Overview of comprehensive sexuality education status in Georgia, Kyrgyzstan, the Republic of Moldova and Tajikistan. In:https://eeca.unfpa.org/sites/default/files/pub-pdf/2024-08/SERAT_4%20country_Summary_V4.pdf. Accessed: 10 February 2025
7. Kapella OB, Laura (2017) Training matters: A framework for core competencies of sexuality educators. In, Colognehttps://whocc.bioeg.de/fileadmin/user_upload/BZgA_TrainingMattersFramework_EN.pdf. Accessed: 25 July 2025
8. Goldfarb ES, Lieberman LD (2021) Three Decades of Research: The Case for Comprehensive Sex Education. J Adolesc Health 68:13-27. 10.1016/j.jadohealth.2020.07.036
9. Haberland N, Rogow D (2015) Sexuality education: emerging trends in evidence and practice. J Adolesc Health 56:S15-21. 10.1016/j.jadohealth.2014.08.013
10. Kantor LM, Lindberg L (2020) Pleasure and Sex Education: The Need for Broadening Both Content and Measurement. Am J Public Health 110:145-148. 10.2105/ajph.2019.305320
11. Ketting E, Friele M, Michielsen K (2016) Evaluation of holistic sexuality education: A European expert group consensus agreement. Eur J Contracept Reprod Health Care 21:68-80. 10.3109/13625187.2015.1050715
12. Michielsen K, Ivanova O (2022) Comprehensive sexuality education: why is it important? In: https://www.europarl.europa.eu/RegData/etudes/STUD/2022/719998/IPOL_STU(2022)719998_EN.pdf. Accessed: 04.12.2025
13. Gayles J, Yahner M, Barker KM et al. (2023) Balancing Quality, Intensity and Scalability: Results of a Multi-level Sexual and Reproductive Health Intervention for Very Young Adolescents in Kinshasa. J Adolesc Health 73:S33-s42. 10.1016/j.jadohealth.2023.02.001

14. Woog VKgA (2017) The Sexual and Reproductive Health Needs of Very Young Adolescents In Developing Countries. In:https://www.guttmacher.org/fact-sheet/srh-needs-very-young-adolescents-in-developing-countries. Accessed: 25 September 2024
15. Igras SM, Macieira M, Murphy E, Lundgren R (2014) Investing in very young adolescents' sexual and reproductive health. Glob Public Health 9:555-569. 10.1080/17441692.2014.908230
16. Anonymous (2020) Executive Summary. Very young adolescent sexual and reproductive health landscape analysis. In:https://resourcecentre.savethechildren.net/pdf/vya_landscape_final.pdf. Accessed: 21 August 2024
17. Anonymous (2024) Building strong foundations. What is foundational education for health and well-being? In:https://unesdoc.unesco.org/ark:/48223/pf0000389751?posInSet=1&queryId=2802c2d9-c9fe-4f10-89a9-23f4c7d47cfc. Accessed: 25 September 2024
18. Robinson D, MacLaughlin V, Poole J (2019) Sexual health education outcomes within Canada's elementary health education curricula: A summary and analysis. The Canadian Journal of Human Sexuality 28:1-14. 10.3138/cjhs.2018-0036
19. Venketsamy T, Kinear J (2020) Strengthening comprehensive sexuality education in the curriculum for the early grades. South African Journal of Childhood Education 1010.4102/sajce.v10i1.820
20. Cacciatore R, Öhrmark L, Kontio J et al. (2024) What do 3–6-year-old children in Finland know about sexuality? A child interview study in early education. Sex Education 24:291-310. 10.1080/14681811.2023.2188182
21. Cacciatore RS, Ingman-Friberg SM, Lainiala LP, Apter DL (2020) Verbal and Behavioural Expressions of Child Sexuality Among 1-6-Year-Olds as Observed by Daycare Professionals in Finland. Arch Sex Behav 49:2725-2734. 10.1007/s10508-020-01694-y
22. Anonymous SEL in the School. A systematic approach integrates SEL across all key settings where students live and learn. In: Collaborative for Academic, Social and Emotional Learning, Chicago https://casel.org/systemic-implementation/sel-in-the-school/. Accessed: 04.12.2025
23. Fraguas D, Díaz-Caneja CM, Ayora M et al. (2021) Assessment of School Anti-Bullying Interventions: A Meta-analysis of Randomised Clinical Trials. JAMA Pediatr 175:44-55. 10.1001/jamapediatrics.2020.3541
24. Gaffney H, Farrington D, Ttofi M (2019) Examining the Effectiveness of School-Bullying Intervention Programmes Globally: a Meta-analysis. International Journal of Bullying Prevention 110.1007/s42380-019-0007-4
25. Anonymous (2022) What works to prevent online violence against children? Executive Summary. In, Genevahttps://iris.who.int/server/api/core/bitstreams/abd8db20-9394-4f4f-9e82-b73933fc3a31/content. Accessed: 25 September 2024
26. Trikic ZA, Ayça (2023) Supporting Families for Gender-Transformative Parenting. In, New Yorkhttps://www.unicef.org/media/134441/file/Gender_Transformative_Parenting_Resource_Modules.pdf. Accessed: 25 September 2024

27. De La Rue L, Polanin J, Espelage D, Pigott T (2014) School-Based Interventions to Reduce Dating and Sexual Violence: A Systematic Review. Campbell Systematic Reviews 10:1-110. 10.4073/csr.2014.7
28. Cherewick M, Lebu S, Su C, Richards L, Njau P, Dahl R (2021) Promoting gender equity in very young adolescents: targeting a window of opportunity for social emotional learning and identity development. BMC Public Health 2110.1186/s12889-021-12278-3
29. Durlak J, Weissberg R, Dymnicki A, Taylor RD, Schellinger K (2011) Enhancing students' social and emotional development promotes success in school: Results of a meta-analysis. Child Development 82:474-501.
30. Kågesten A, van Reeuwijk M (2021) Healthy sexuality development in adolescence: proposing a competency-based framework to inform programmes and research. Sex Reprod Health Matters 29:1996116. 10.1080/26410397.2021.1996116
31. Cacciatore R, Korteniemi-Poikela E, Kaltiala R (2019) The Steps of Sexuality—A Developmental, Emotion-Focused, Child-Centred Model of Sexual Development and Sexuality Education from Birth to Adulthood. International Journal of Sexual Health 31:1-20. 10.1080/19317611.2019.1645783
32. van der Doef S, Reinders J (2018) Stepwise sexual development of adolescents: the Dutch approach to sexuality education. Nat Rev Urol 15:133-134. 10.1038/nrurol.2018.3
33. Fortenberry JD (2013) Puberty and adolescent sexuality. Horm Behav 64:280-287. 10.1016/j.yhbeh.2013.03.007
34. Fine M (1988) Sexuality, Schooling, and Adolescent Females: The Missing Discourse of Desire. Harvard Educational Review 58:29-54. 10.17763/haer.58.1.u0468k1v2n2n8242
35. Garland-Levett S, Allen L (2018) The Fertile, Thorny, and Enduring Role of Desire and Pleasure in Sexuality Education. In: Lamb S, Gilbert J (eds) The Cambridge Handbook of Sexual Development: Childhood and Adolescence. Cambridge University Press, Cambridge, p 521-536
36. Landi N (2017) 'Pleasure Is Not in the Science Programme!': When Anthropology Engages with Sex Education for Teenagers. https://anthropologymatters.com/index.php/anth_matters/article/view/479 Accessed: 04.12.2025
37. Singh A, Both R, Philpott A (2021) 'I tell them that sex is sweet at the right time' - A qualitative review of 'pleasure gaps and opportunities' in sexuality education programmes in Ghana and Kenya. Glob Public Health 16:788-800. 10.1080/17441692.2020.1809691
38. Allen L, Carmody M (2012) 'Pleasure Has No Passport': Re-visiting the Potential of Pleasure in Sexuality Education. Sex Education 12:455-468. 10.1080/14681811.2012.677208
39. Nahar P, Reeuwijk M, Reis R (2013) Contextualising sexual harassment of adolescent girls in Bangladesh. Reproductive health matters 21:78-86. 10.1016/S0968-8080(13)41696-8
40. Ford J, Vargas E, Finotelli Jr Í et al. (2019) Why Pleasure Matters: Its Global Relevance for Sexual Health, Sexual Rights and Wellbeing. International Journal of Sexual Health 31:1-14. 10.1080/19317611.2019.1654587

41. Philpott A, Larsson G, Singh A, Zaneva M, Gonsalves L (2021) How to Navigate a Blindspot: Pleasure in Sexual and Reproductive Health and Rights Programming and Research. Int J Sex Health 33:587-601. 10.1080/19317611.2021.1965690
42. Zaneva M, Philpott A, Singh A, Larsson G, Gonsalves L (2022) What is the added value of incorporating pleasure in sexual health interventions? A systematic review and meta-analysis. PLoS One 17:e0261034. 10.1371/journal.pone.0261034
43. Castellanos-Usigli A, Braeken-van Schaik D (2019) The Pleasuremeter: exploring the links between sexual health, sexual rights and sexual pleasure in sexual history-taking, SRHR counselling and education. Sex Reprod Health Matters 27:1-3. 10.1080/26410397.2019.1690334
44. Braeken DC-U, Antón (2018) Sexual Pleasure. The forgotten link in sexual and reproductive health and rights. Training Toolkit. In: Global Advisory Board (GAB) for Sexual Health and Wellbeinghttps://www.gab-shw.org/media/1024/gab_sexualpleasuretrainingtoolkit_final_webversion_withhyperlinks_updateapril2019.pdf. Accessed: 01.10.2024
45. Gruskin S, Yadav V, Castellanos-Usigli A, Khizanishvili G, Kismödi E (2019) Sexual health, sexual rights and sexual pleasure: meaningfully engaging the perfect triangle. Sex Reprod Health Matters 27:1593787. 10.1080/26410397.2019.1593787
46. Haberland NA (2015) The case for addressing gender and power in sexuality and HIV education: a comprehensive review of evaluation studies. Int Perspect Sex Reprod Health 41:31-42. 10.1363/4103115
47. Farrelly N, Barter C, Stanley N (2022) Ready for Relationships Education? Primary school children's responses to a Healthy Relationships programme in England. Sex Education 23:1-17. 10.1080/14681811.2022.2052834
48. Barrense-Dias Y, Akre C, Surís JC et al. (2020) Does the Primary Resource of Sex Education Matter? A Swiss National Study. J Sex Res 57:166-176. 10.1080/00224499.2019.1626331
49. Neary A (2023) Intersections of age and agency as trans and gender diverse children navigate primary school: listening to children in (re)considering the potential of sexuality education. Sex Education 24:1-15. 10.1080/14681811.2023.2238634
50. Michielsen K, Brockschmidt L (2021) Barriers to sexuality education for children and young people with disabilities in the WHO European region: a scoping review. Sex Education 21:1-19. 10.1080/14681811.2020.1851181
51. Riggs D, Bartholomaeus C (2017) Transgender young people's narratives of intimacy and sexual health: implications for sexuality education. Sex Education 18:1-15. 10.1080/14681811.2017.1355299
52. Ullman J (2016) Teacher positivity towards gender diversity: exploring relationships and school outcomes for transgender and gender-diverse students. Sex Education 17:1-14. 10.1080/14681811.2016.1273104
53. Bengtsson J, Bolander E (2019) Strategies for inclusion and equality – 'norm-critical' sex education in Sweden. Sex Education 20:1-16. 10.1080/14681811.2019.1634042
